# Editorial: Application of New Technologies in the Treatment of Substance Use Disorders

**DOI:** 10.3389/fpsyt.2021.626357

**Published:** 2021-02-12

**Authors:** Kai Zhang

**Affiliations:** Department of Psychiatry, Chaohu Hospital, Anhui Medical University, Hefei, China

**Keywords:** new technologies, tobacco use disorder, transcranial magnetic stimulation, substance use disorder, smartphone application, working memory

Substance use disorders (SUDs)—a social, economic, and medical burden—are a global problem. Over time, the number of people with SUDs has increased significantly. SUDs are usually resolved with psychotherapy, but when necessary, pharmacotherapy is also required. Most of the time, however, these methods are not always effective and this poses a significant challenge for developing more effective therapies. As technology advances, it brings new hope for the treatment of SUDs.

Bu et al. found that the low-theta coherence network could significantly predict changes in individuals' cigarette cravings. Thus, the low-theta EEG coherence network in smokers' brains might be a biomarker of smoking cue reactivity and predict addiction behavior.

Tobacco use disorder (TUD) is currently treated with nicotine replacement therapy. This treatment is effective, but relapses are common, especially in the face of various TUD cue stimuli. In their case report, Peechatka et al. found that nicotine replacement therapy, coupled with nicotine free electronic cigarette use, was a promising option in preventing the relapse of TUD. However, these results are yet to be validated by a large randomized controlled study.

Transcranial magnetic stimulation (TMS), a new technology developed in recent years, has been widely used to treat mental illness. Studies have shown that repeated TMS is equally useful for treating SUDs (1). Newman-Norlund et al. found that theta-burst repeated TMS to the inferior frontal gyrus promotes nicotine addiction inhibitory control.

In recent years, the number of people with methamphetamine use disorders (MUD) in China has increased annually (2). The proportion of people with MUD has surpassed that of users of traditional drugs such as heroin. The treatment of MUD is also a significant challenge for the Chinese government. Wang et al. proposed a new approach for the treatment of MUD; they aimed to test the efficacy of paliperidone extended-release in decreasing methamphetamine use and reducing psychotic symptoms in methamphetamine-dependent patients after detoxification. Paliperidone administration resulted in a better retention rate and lowered psychotic symptom relapse among patients with psychotic disorders using methamphetamine. Based on more evidence from clinical studies, paliperidone, an antipsychotic, may be used to treat MUD in people with concomitant psychotic disorder in the future. Chen et al. have suggested psychological treatment for SUDs, using an autonomic progress bar to motivate. Their study's novel progress-bar tool effectively motivated treatment completion. It was also effective in forecasting continually negative urine test results. The tool's free open-source code makes it easy to implement in many substance-treatment services.

The increasing use of smartphones has accelerated the development and research of mobile phone-based interventions for SUD therapy (3–5). Smartphone applications offer various features with advanced software capabilities that can enhance chronic disease management. Clinical studies have increasingly examined whether smartphone applications can offer effective intervention in alcohol use disorders (AUD). Berman et al. conducted a pilot randomized controlled trial to evaluate the clinical impact of TeleCoach™, a web-based skills training smartphone application, on adult participants with AUD recruited from the internet in Sweden. After a 6-week intervention, there were significant within-group effects on alcohol consumption, but there were no significant between-group differences.

Why can mobile applications reduce alcohol use in people with AUD? It has been suggested that working memory training mobile phone applications may have a therapeutic effect by improving working memory in patients with SUD (6). Working memory training (WMT) is a promising way to improve working memory. A systematic review article by Brooks et al. showed that repeated WMT reduces brain activation in the frontoparietal and striatal networks, reflecting increased neural circuitry efficiency via myelination and functional connectivity changes. WMT could be utilized as a promising, effective, and non-invasive intervention for working memory deficits to treat impulse and affective control problems in people with SUDs.

## Author Contributions

KZ contributed to the writing of the Editorial.

## Conflict of Interest

The author declares that the research was conducted in the absence of any commercial or financial relationships that could be construed as a potential conflict of interest.
